# Representative Proteomes: A Stable, Scalable and Unbiased Proteome Set for Sequence Analysis and Functional Annotation

**DOI:** 10.1371/journal.pone.0018910

**Published:** 2011-04-27

**Authors:** Chuming Chen, Darren A. Natale, Robert D. Finn, Hongzhan Huang, Jian Zhang, Cathy H. Wu, Raja Mazumder

**Affiliations:** 1 Center for Bioinformatics and Computational Biology, University of Delaware, Newark, Delaware, United States of America; 2 Protein Information Resource, Department of Biochemistry and Molecular & Cellular Biology, Georgetown University Medical Center, Washington, D. C., United States of America; 3 Janelia Farm Research Campus, Howard Hughes Medical Institute, Ashburn, Virginia, United States of America; Deutsches Krebsforschungszentrum, Germany

## Abstract

The accelerating growth in the number of protein sequences taxes both the computational and manual resources needed to analyze them. One approach to dealing with this problem is to minimize the number of proteins subjected to such analysis in a way that minimizes loss of information. To this end we have developed a set of Representative Proteomes (RPs), each selected from a Representative Proteome Group (RPG) containing similar proteomes calculated based on co-membership in UniRef50 clusters. A Representative Proteome is the proteome that can best represent all the proteomes in its group in terms of the majority of the sequence space and information. RPs at 75%, 55%, 35% and 15% co-membership threshold (CMT) are provided to allow users to decrease or increase the granularity of the sequence space based on their requirements. We find that a CMT of 55% (RP55) most closely follows standard taxonomic classifications. Further analysis of this set reveals that sequence space is reduced by more than 80% relative to UniProtKB, while retaining both sequence diversity (over 95% of InterPro domains) and annotation information (93% of experimentally characterized proteins). All sets can be browsed and are available for sequence similarity searches and download at http://www.proteininformationresource.org/rps, while the set of 637 RPs determined using a 55% CMT are also available for text searches. Potential applications include sequence similarity searches, protein classification and targeted protein annotation and characterization.

## Introduction

There are several ongoing efforts aimed at reducing the redundancy in protein sequence space. Examples of such efforts include National Center for Biotechnology Information's non-redundant protein database (NCBI-nr) and UniProt Consortium's UniRef (UniProt Reference Clusters) [1]. NCBI-nr clusters identical proteins from the same organism whereas UniRefs provide clustered sets of sequences at several resolutions (100%, 90% and 50%). Both methods hide redundant sequences while providing ways to access them if needed. These databases are widely used for various applications, but may not always be optimal for functional annotation and protein classification with the ever-increasing target sequence space [2]. Another approach is to use only complete proteome sets. NCBI's RefSeq project [3] and UniProtKB complete proteome projects (http://www.uniprot.org/taxonomy/complete-proteomes) provide users with the ability to perform analyses or create protein families using the limited sequence space of complete proteomes. A major advantage is that orthologs and paralogs can be more precisely discerned. However, since the overwhelming majority of new sequences derive from completely-sequenced genomes ((with more than 1000 proteomes already sequenced and 1000 s more to come within the next year or so (http://www.genomesonline.org/gold_statistics.htm)), this approach offers limited benefit over using the entire sequence space. A related approach is to select proteins from a subset of genomes and deal exclusively with those. Efforts are already underway that manually designate some genomes as Reference Genomes, such as Gene Ontology Reference Genomes [4] and Quest for Orthologs [5]. These chosen genomes were selected either because of model organism status and/or because of their position in the taxonomic tree; how well these represent sequence space was not tested.

The critical question is how to select the proteomes to be included in such a standard set to achieve reduced sequence space, while retaining the majority of the annotation and diversity of sequences. How to choose such proteomes should be based on the purpose for which the final set is intended. Because requirements may vary, there has to be an objective yet flexible way of obtaining representative proteomes at different levels of granularity. For example, for hierarchical protein family classification [6] and functional annotation one may choose a larger or smaller set of representative proteomes depending on the phyletic distribution of the protein family members and sequence variation. For sequence similarity searches one could choose to use a set of representative proteomes as an initial filter prior to comprehensive search against the entire protein space. Thus, the following criteria arise: 1) Each RP member must be good representatives (in an evolutionary context) of the proteomes that are not included in the reduced set; 2) The RP member should be the most functionally characterized/annotated member of the group; and 3) The RPs at different thresholds should be hierarchical. That is – if a proteome is a representative at a lower CMT (such as RP15), it should also be a representative at a higher CMT (such as RP75). This will allow users to select whichever set suits the intended purpose.

Keeping the above criteria in mind we have developed an algorithm (see materials and methods) that can reliably and quickly calculate a hierarchical set of RPs at different thresholds for cellular organisms (archaea, bacteria, and eukaryota).

## Materials and Methods

### Data Sources

Unless otherwise noted the source sequences for this representative proteome project are from UniProtKB release 2010_09 [7]. Proteomes missing the “complete proteome” keyword in UniProtKB (as is the case for some GO Reference Genomes) were retrieved from Ensembl [8]. Protein sequence clusters were from UniRef50 which covers all UniProtKB sequences [1]. The list of characterized proteins (29,607 unique proteins with 29,632 unique references; see Table S1 – tab-delimited: UniProtKB accession, UniProtKB identifier, protein name, PubMed identifier, paper title) was created as follows: curated literature references from two sources were compared: “RP” lines in UniProtKB/Swiss-Prot entries and literature evidence from the Gene Ontology Annotation file (GAF). PubMed identifiers found in both data sources for the same entry indicates that the protein therein was experimentally characterized, as confirmed by independent curators. The GAF file used for Gene Ontology annotations (ftp.geneontology.org/pub/go/gene-associations/submission gene_association.goa_uniprot.gz) was downloaded on 24-August-2010. The UniProtKB/Swiss-Prot file used for literature information (ftp.uniprot.org/pub/databases/uniprot/knowledgebase uniprot_sprot.dat.gz) is from release 2010_09.

### Finding the representative proteomes using UniRef50

**Figure 1:**
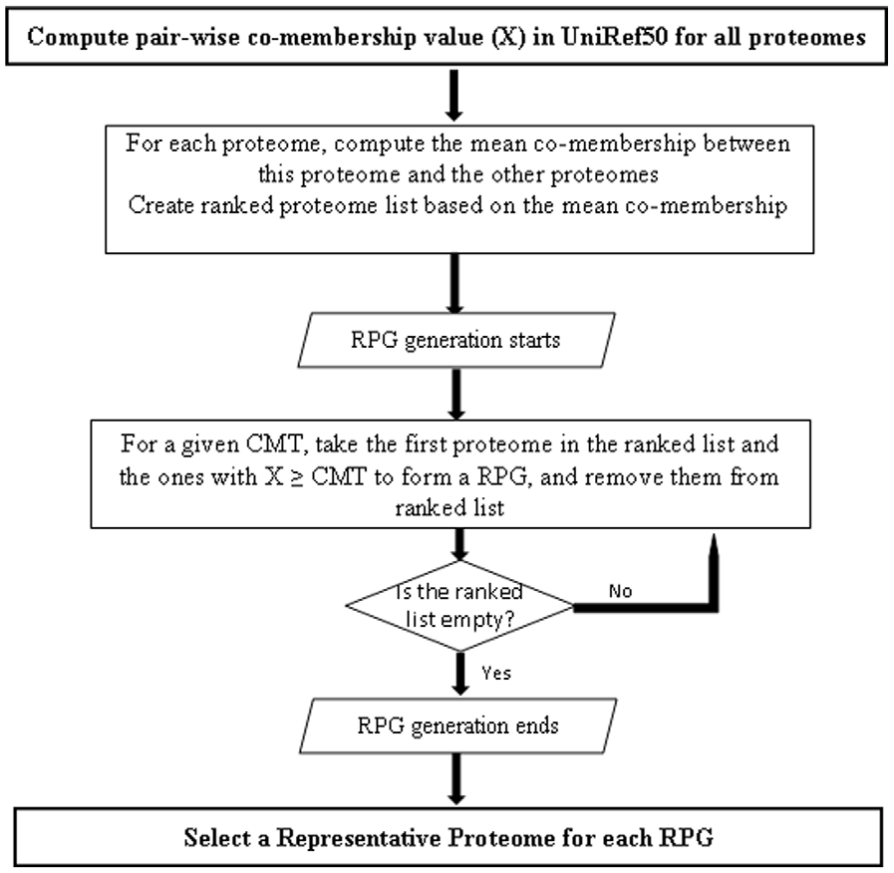
Flow chart of the method used to select Representative Proteomes. For details please see materials and methods section.

An overview of the algorithm used to find the representative proteomes is shown in Figure 1 and described in detail below.

### Co-membership of two proteomes in UniRef50

Given two proteomes A and B, their co-membership in the UniRef50 is measured by the following value:
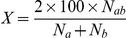
where


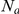
 is the number of UniRef50s containing a protein from proteome A,


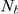
 is the number of UniRef50s containing a protein from proteome B,


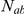
 is the number of UniRef50s containing a protein from both proteomes.

Given a co-membership threshold 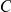, if 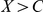, the two proteomes are grouped together.

### Compute Representative Proteome Group (RPG)

**Step 1.** Given a list of complete proteomes, the co-membership value (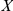) for each pair of proteomes is calculated. Then for each proteome, the mean co-membership is computed, which is the average 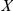 value between this proteome and the other proteomes.

**Step 2.** The list of proteomes is ranked according to mean co-membership.

**Step 3.** The first proteome from the ranked proteomes list is taken and the above method is applied to generate the first group.

**Step 4.** The proteomes in the first group are removed from the list.

Steps 3 and 4 are repeated to generate the rest of the RPGs until the ranked proteomes list is empty or the remaining RPGs consist of a single proteome. In the case of the latter, each single proteome becomes the RP.

### Select Representative Proteome from a RPG

Proteomes in each RPG are ranked to facilitate the selection of a representative proteome for the group. The proteomes are ranked as follows:

1. Number of unique PubMed references with some type of functional characterization from all the proteins in the proteome, excluding papers annotated with the phrases “LARGE SCALE ANALYSIS”, or “COMPLETE GENOME” in UniProtKB RP lines,
2. Number of unique PDB database cross-references from all the proteins in the proteome,
3. Number of UniProtKB/Swiss-Prot entries in the complete proteome,
4. Number of entries in the complete proteome.

A Proteome Priority Score (PPS) is used for the ranking:

PPS = 1000(1+NS_PMID_)+100(1+NS_PDB_)+10(1+NS_UniProtKB/Swiss-Prot_)+(1+ NS_Entry_)

where the normalized score (NS) for each item is obtained by dividing the raw number by the maximum observed for each item (NS will thereby vary from 0 to 1).

Given a Representative Proteome Group consisting of a list of complete proteomes, the proteome with the greatest PPS is selected as the Representative Proteome for the group. In the future the PPS will include an additional weighting factor that favors previously determined RPs to ensure stability of this set, and any replacement of an existing RP will be supervised by a curator. To evaluate the results and find a default threshold value ideal for protein classification and BLAST search, RPGs were computed at 10–80 CMT with 5% increment.

### Addition of Gene Ontology Reference Genomes

The GO Reference Genome project is committed to providing comprehensive GO annotations for the human genome, and eleven important model organisms: *Arabidopsis thaliana*, *Caenorhabditis elegans*, *Danio rerio*, *Dictyostelium discoideum*, *Drosophila melanogaster*, *Escherichia coli*, *Gallus gallus*, *Mus musculus*, *Rattus norvegicus*, *Saccharomyces cerevisiae*, and *Schizosaccharomyces pombe*. Collectively those twelve species are referred to as the “GO Reference Genomes” [4]. All downloadable sequence datasets contain these model organisms as RPs irrespective of the automatic selection based on RPGs (depending on the CMT, some of the Reference Genome organisms would not otherwise have been RPs, and the four species lacking the ‘complete proteome’ keyword in UniProt–*Danio rerio, Gallus gallus, Mus musculus, Rattus norvegicus*–were not considered in the RPG calculations). Proteomes of GO Reference Genomes that are not available in UniProt were obtained from Ensembl and provided in the sequence download files. The Ensembl set was filtered to yield only one entry per gene. This was done in the following manner. 1) Map every UniProtKB/Swiss-Prot and UniProtKB/TrEMBL entry for these organisms to an Ensembl protein and gene. 2) Go through the UniProtKB/Swiss-Prot entries, and mark as “already found” any Ensembl protein ID and corresponding gene ID. List the UniProtKB/Swiss-Prot accessions. 3) Go through the UniProtKB/TrEMBL entries, skipping those that map to already-found Ensembl protein or gene ID (based on the previous UniProtKB/Swiss-Prot mapping). For the remaining ones, order the proteins based on size, and mark the Ensembl protein and gene IDs as “already found” after taking the longest one for each gene. 4) Go through the Ensembl set and filter as done in step 3. The resulting list closely represents one-entry-per-gene for each organism, retaining UniProtKB/Swiss-Prot and UniProtKB/TrEMBL as much as possible. When these proteomes get the “complete proteome” keyword in UniProtKB then this pipeline will be discontinued as the preference is to get all sequences from UniProtKB directly.

## Results

This study aims to obtain a set of representative proteomes that would serve as the basis for protein sequence search and classification and targeted protein annotation and characterization. This aim leads to two major specifications: First, the initial determination of representatives should be based on sequence considerations alone; and second, the final set of representatives should include model organism proteomes.

Taking into account the first specification, we based our selection procedure on UniRefs, which consist of clustered sets of sequences from the UniProt Knowledgebase (UniProtKB) and selected UniProt Archive records [1]. As part of the UniProt production pipeline, UniRefs are updated every four weeks in conjunction with the UniProtKB release. UniRef50 was chosen to calculate co-membership because it is computationally generated using UniProtKB proteins in such a way that the clusters are more likely to be tight clusters of orthologs and inparalogs. As a result, it tends to group proteins that are from relatively close genomes to the exclusion of more distant ones, which is exactly what is desired to create the RPGs.

Taking into account the second specification, we supplemented the representatives selected on the basis of sequence with additional proteomes based on the Gene Ontology Reference Genome Project.

### Evaluation of RPGs at different co-membership thresholds (CMTs)

The co-membership threshold (CMT) in UniRef50 used to group two proteomes together was adjusted to provide RPGs which have different reductions in the number of proteomes and sequences. Table 1 shows the summary results for four of the fifteen different thresholds tried. The data show that very low CMTs tend to greatly reduce sequence space at the cost of producing RPGs that contain multiple genera in one RPG. Very high CMTs have a low reduction of sequence space, and may tend to split species from a given genus into multiple RPGs. A threshold of 55% provides a set of Representative Proteomes (RP55) that most closely resembles what would be obtained by simple selection based on the taxonomy tree (thereby displaying a minimum combined level of split species and merged genera). At this threshold a set of 637 RPGs were generated from 1144 complete proteomes. Only a handful of RPGs have proteomes from multiple genera (nine) and very few species (eleven, based on taxonomy) are present in different RPGs. A further analysis of these species and genera that group differently reveals that a majority of them have well known naming discrepancies. To assist users who might prefer more or less granularity, data at three other thresholds (RP15, RP35, and RP75) are also available for download (Table S2 provides statistics on the cluster sizes for all four RP sets).

**Table 1:** Representative Proteomes computed at different thresholds.

Threshold	#RPG	%reduction in #proteomes	%reduction in #sequences	%species in multiple RPGs	%RPG has multiple genus proteomes	RP Sequence Coverage (%)	RP UniRef50 Coverage (%)
15	278	75.6993	53.5041	0.3713	31.6547	25.1806	51.2873
35	499	56.3811	45.5558	0.8663	6.6132	43.3308	75.8166
55	637	44.3182	37.9064	1.3614	1.2559	56.8638	88.2188
75	763	33.3042	30.3225	3.3416	0.3932	67.1059	93.1352

Based on UniProt: 2010_09; # of organisms: 1144; # of species: 808; # of genus: 453; # of sequences: 4335476; # of UniRef50 clusters: 1566987.

To guarantee the proteomes grouped together stay together even at a lower CMT we use a top-down approach to compute the RPGs. Only RPs from the previous iteration are used to create the ranked proteomes list (i.e. RP75 proteomes were used to create the RP55 set which in turn was used to create the RP55 set and so on). The members of RPGs from the previous iteration are added to the new RPGs accordingly. The remaining analyses focus on the RP55 set.

### Consistency of RPGs and RPs

**Figure 2:**
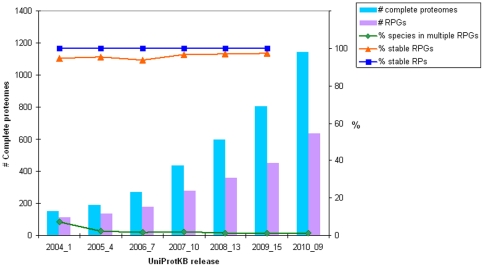
Stability and characteristics of RP55. RPGs and RPs were determined for previous releases of UniProtKB. Histograms show the growth in the number of RPs relative to the number of complete proteomes. The percentage of species with strains found in multiple RPGs is given by the green line, while the percentages of RPGs and RPs that remained unchanged between the indicated release and the 2010_09 release are given by the orange and blue lines, respectively.

RPs are desired to be stable. As a measure of stability RPGs were computed for previous releases of UniProtKB to evaluate the consistency of the RPGs over time. Figure 2 shows the statistics using a 55% CMT. For illustration purposes, only one release per year is shown. The percentage of species in multiple RPGs is low–less than 2% since 2006–indicating that the quality of the clustering is consistently good over increasing sequence space. To estimate the stability of the RPGs, we traced all RPGs in every year and checked for membership variation. The results indicate 94% or more of the RPGs introduced over time are stable. Despite the six-fold increase in number of complete proteomes, of the 116 RPGs present in the initial 2004 set, we find that 99.3% of the groups contain the same set of member proteomes in 2010 (albeit with additional member proteomes). Furthermore, all RPs remained as such from 2004 through 2010. We additionally tested the consistency of RPs using different sorting methods such as PPS, size of proteome and at random (no sorting) using the UniProtKB 2010_09 release. The different methods give very similar clustering–more than 98% of the proteome groups consist of the same set of members and all RPs are the same (data not shown).

### Representative Proteome coverage and size reduction

The major objectives in producing the RP set are to reduce the sequence space while preserving as much as possible both sequence representation and annotation content; here we evaluate how representative this reduced set is of the complete set.

First, we calculate the extent that sequence space is reduced. The RP15 (threshold 15%), RP35, RP55 and RP75 sets yield a database size reduction of 53, 45, 37 and 30%, respectively, with respect to the source sequence set (of complete proteomes). In terms of UniProtKB overall the reduction is even more pronounced. For example, for RP55 the size reduction from UniProtKB is almost 80%. It is expected that the size reduction will increase significantly over time as more and more related genomes are sequenced. Indeed, over the time period examined, though both the number of complete proteomes and RPGs are increasing exponentially, the number of RPGs is doing so at a lower rate, and may be showing signs of leveling off (Figure 2 and data not shown).

Next, we analyze how many InterPro [9] profiles contained at least one protein from the RP set. Since the RPs were drawn solely from archaea, bacteria, and eukaryota, we first calculated the number of InterPro profiles that were found within only those kingdoms (excluding, for example, profiles that hit only virus sequences) and searched this subset of profiles against the RP55 set. Over 95% are found in the RP set (19,016 out of 19,715). The nature of the missed InterPro profiles was examined. A large number (∼150) were predominantly virus-specific families that happened to have a small number of non-virus sequences. Spot checks indicate that these non-viral sequences are due to source contamination, or a viral/phage sequence that was integrated into a genome. Another reason for missed InterPro entries seems to be that they are either short (with poor separation of signal and noise due to insufficient information content in the profile) or lineage-specific. For example, there were a number of toxins or anti-toxins (IPR020475, Bibrotoxin/Sarafotoxin-D; IPR016330, Neurotoxin_III_Actiniidae), restriction endonucleases (IPR021108, Restrct_endonuc_II_BpuJI_N; IPR019067, Restrct_endonuc_II_MamI), and hormones (IPR020382, Androgenic_gland_hormone_art; IPR016058, Pheromone_Er1_protoz), and several fish-specific families (IPR020410, Interleukin-15_fish; IPR020691, Tyr_kinase_rcpt_erbB3_fish). InterPro covers more than 75% of all UniProtKB sequences, and this number holds true for the archaea, bacteria and eukaryota subset (75.6%). The percent of RP proteins covered by InterPro was calculated, and again the number was found to exceed 75% (76.7%). This implies that the representative set is neither over- nor under-populated with lineage-specific proteins.

We next evaluate how well the RP55 set retains the information content of the full set by counting how many of the approximately 30,000 characterized proteins (see Materials and Methods) are present. Approximately 93% are found in the RP set. The RP55 set also retains information content by other measures. For example, despite reducing the size to 20% of UniProtKB, the 2,415,222 proteins in the RP55 set contain nearly 45% of all UniProtKB/Swiss-Prot entries (230,889 of 519,348) and over 45% of all structurally-characterized proteins (13,453 of 28,770 with PDB cross-references). Combining the coverage, information content, and size reduction data given above, we conclude that the RP set indeed achieves the objectives of reduced size without significant loss of similar-sequence coverage and functional annotation information.

### Representative Proteome similarity searches

The suitability of the RPs for sequence similarity searches was tested. Performing searches against the entire protein space is time consuming. For example, the computation time to perform an all-against-all BLAST search of the entire UniProtKB is approximately 4.1 CPU years. Using RP55, the time to perform an equivalent BLAST search is only 0.23 CPU years, a nearly 20-fold reduction. This time advantage is over and above other savings, such as time required to post-process the results or other overhead such as disk space required to store the results.

**Figure 3:**
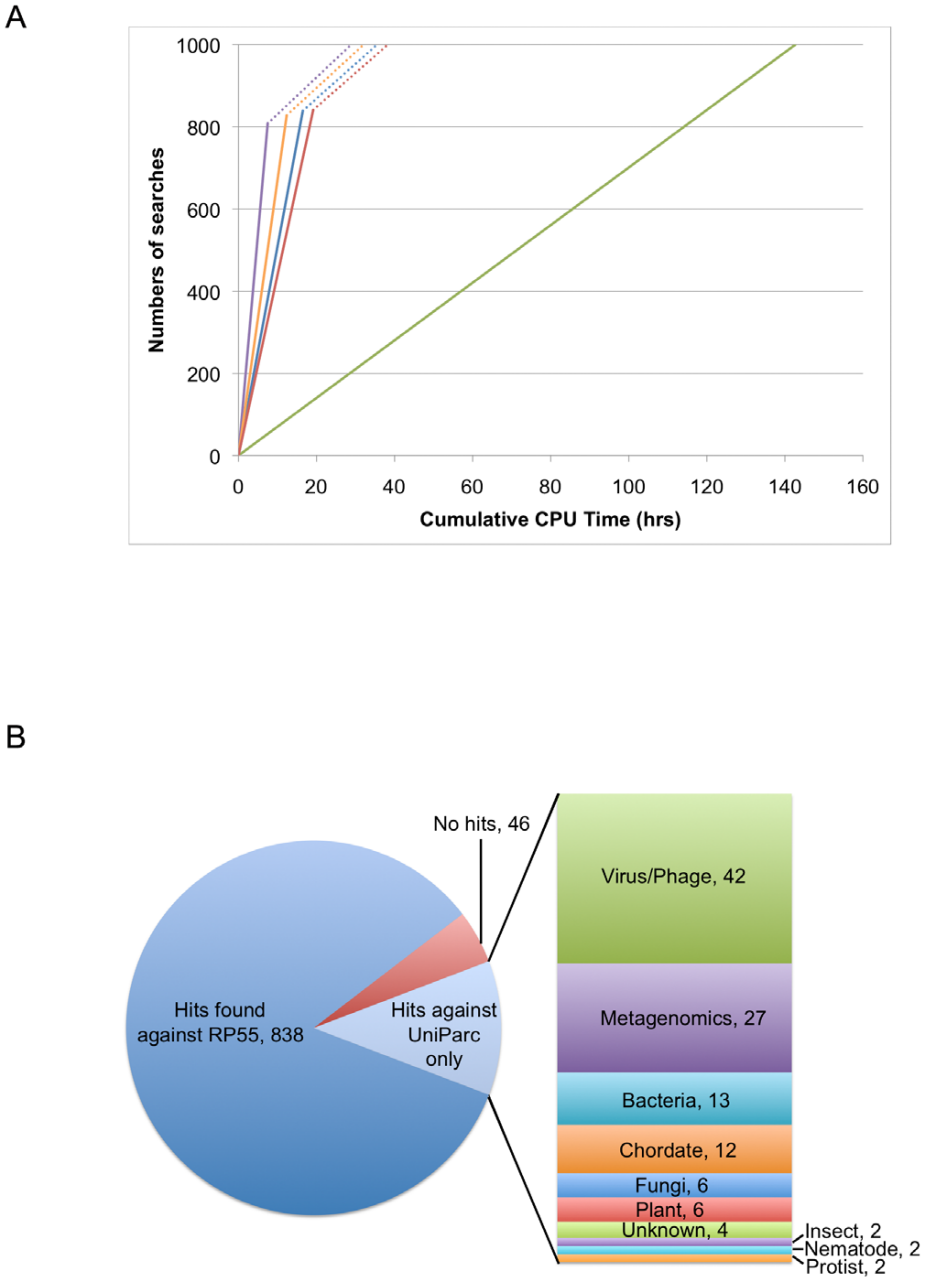
Sequence similarity searches against Representative Proteome sets. 3a) Time required to perform phmmer searches on 1000 randomly chosen UniParc sequences against RP15 (purple), RP35 (orange), RP55 (blue) and RP75 (red) or UniParc (green solid lines). The subset of sequences with no Representative Proteome (RP) hits were searched against the whole of UniParc and the two search times where summed (broken lines). 3b) Taxonomic breakdown of the subset of sequences without RP hit.

To further understand how RPs could be used to perform similarity searches, we analyzed 1000 randomly chosen sequences from UniParc [10] (release 2010_09) and searched these query sequences against four %CMT RP sets as target databases using phmmer (a HMM based method for searching a single sequence against a target database (http://hmmer.janelia.org/)), using an E-value threshold of 0.01 and default phmmer parameters. If a query sequence did not match any sequences in the target RP database, that query sequence was then re-searched using UniParc as the target database and the search times summed (dashed lines in Figure 3a). As a baseline we also searched these 1000 sequences against UniParc. The results are summarized in Figure 3a. Searching the 1000 sequences against UniParc alone took just over 140 CPU hours (using a 2.66 GHz Intel processor), whereas all of the searches against RP databases plus complete database searches for non-matching query sequences (four independent searches of 1000 sequences each) took under 40 CPU hours, a saving of 100 CPU hours. In every case, over 80% of the query sequences were matched when using RP as a target database, regardless of the threshold used. In the case of RP55, 838 query sequences matched one or more sequences. The remaining 162 sequences were then searched against UniParc. It was not possible to obtain a significant match using the defined search parameters for 46 of these. The non-matching query sequences were short (less than 30 amino acids), often with composition bias. The remaining 116 sequence all had matches against UniParc, but not RP55. These query sequences, with an average length of 175 amino acids, are shorter than the average UniProtKB or query sequence length. In addition, they predominantly came from sources that are not expected to be represented in RP55 (Figure 3b), namely, viruses and metagenomics sequences (where the source organism is unknown).

To analyze the nature of the matches against RP a little further and to provide an insight as to whether the match was a ‘good’ match, we took the list of hits and compared them to the corresponding hits when the whole of UniParc was searched. We then used two different ways to test if the matches were good: by scores and by coverage of the query sequence (Table 2). Hits identical to the query sequence were ignored; therefore 912 queries were compared between RP55 and UniParc (46 queries lacked any hit at all to RP55, and 42 queries only had a significant hit to itself). In the first test, test 1, the hits were classified into the following categories: 1) The RP hit was equivalent to the highest scoring match from UniParc; 2) The ratio of the log of the E-value of the highest scoring RP hit and the highest scoring UniParc hit was less than 0.5 (these were deemed “good enough” hits); 3) Only the full search provides a ‘good’ scoring match. In the second test, test 2, we looked at the coverage of query sequence between the highest scoring hit from RP compared to the highest scoring hit from UniParc. Again, the hits were classified in to three groups: 1) RP hit was equivalent to highest scoring match from UniParc; 2) The coverage between the query and its highest scoring RP hit and the highest scoring UniParc hit were within 95% (these were deemed “good enough” hits); 3) Only the full search provides a ‘good’ coverage match. The outcomes of both tests were fairly similar, with 60–78% of searches against RP giving the best search result, or at least within close proximity to the best. Thus, RP not only saves time but will also give a ‘good’ hit, when a match is found.

**Table 2:** Assessing Representative Proteomes in different ways (phmmer score and coverage of query sequence in terms of amino acid overlap).

Category	RP15	RP35	RP55	RP75
*Test 1*	*Score (phmmer)*			
RP = UniParc	100	147	182	215
RP good enough	444	468	459	433
Full search only	398	327	301	294
*Test 2*	*Coverage*			
RP = UniParc	100	147	182	215
RP good enough	544	539	526	499
Full search only	298	256	234	228

In a separate attempt, we used the RP set to identify proteins unique to an individual species [11]. A protein was considered to be unique if it 1) does not co-occur with a protein from any other species in a UniRef50 cluster; and 2) does not hit any protein from another species when searched against the RP set using BLAST (e-value cutoff of 1.0×10^−4^); and 3) does not hit any protein from another species when search against UniProtKB using BLAST (e-value cutoff of 1.0×10^−4^). From an initial collection of 1008 bacterial genomes containing 3,334,488 proteins, the UniRef50 filtering step (step 1) resulted in a 79.25% size reduction, BLAST against RP (step 2) reduced the number an additional 72.53%, and BLAST against UniProtKB (step 3) further reduced the size by 14.39%. These data indicate that using the RP set can significantly reduce the search space that is necessary for performing pair-wise sequence similarity searches while providing almost complete coverage.

### Availability and usage

A dedicated web site (http://www.proteininformationresource.org/rps) is available to disseminate the RP and RPGs and related sequence data, including functionalities for sequence searching, data set browsing and file downloading. For the threshold values of 75, 55, 35, and 15, corresponding Representative Proteome Group files are provided in the format as below:

>rp_taxon_id rp_code rp_name taxon_group rp_annotation_score (AS) C(THRESHOLD)

mp_taxon_id mp_code mp_name taxon_group mp_annotation_score (AS) X_to_rp(X)

…

>rp_taxon_id rp_code rp_name taxon_group rp_annotation_score (AS) C(THRESHOLD)

…

Where

rp is Representative Proteome,

mp is Member Proteome in the Representative Proteome Group

An example of Representative Proteome Group is shown below:

>205920 EHRCR Ehrlichia chaffeensis (strain Arkansas) Bac/Alpha-proteo 1111.19332(AS)55(CUTOFF)

269484 EHRCJ Ehrlichia canis (strain Jake) Bac/Alpha-proteo 1111.10824(AS)71.20366(X)

302409 EHRRG Ehrlichia ruminantium (strain Gardel)Bac/Alpha-proteo 1111.10730(AS)64.05622(X)

254945 EHRRW Ehrlichia ruminantium (strain Welgevonden) Bac/Alpha-proteo 1111.12521(AS)65.56531(X)

Also provided are the sequence files in FASTA format for the RP75, RP55, RP35 and RP15 sets. Users can choose to make their own customized RP set by using the taxon-based table or Perl script available via a link from the home page. For example, we suspect that, for some users, the ideal set could be RP75 for Animals + RP55 for other cellular organisms + any missing GO Reference Genomes. Using a higher threshold for animals makes sense because all the phyla of animals appeared within a short time frame which results in closer inter-genera molecular relationships but wide phenotypic characteristics. In the future, viral reference proteomes as defined by UniProt (http://www.uniprot.org/news/2010/07/13/release) will be provided, along with widely requested customized RP sets.

### Representative Proteome availability from the iProClass interface

*i*ProClass is an integrated data-warehouse containing all UniProtKB proteins and additional proteins from NCBI resources [12]. The proteins from the defined representative proteome sets are indexed in *i*ProClass and are available for BLAST searches (http://proteininformationresource.org/rps/blast_rp.shtml). Additionally, all the proteins from the RP55 set can be retrieved from http://proteininformationresource.org/pirwww/search/textsearch.shtml by selecting Rep Proteome and then typing in not null and clicking on Search (2,855,382 proteins at the time of writing this paper). Users can perform additional filtering on the retrieved set by performing Boolean searches using more than 65 fields available from the text search pull down menu. The BLAST and the text search results can be downloaded from the results page for further analysis.

### Browsing the Representative Proteomes

**Figure 4:**
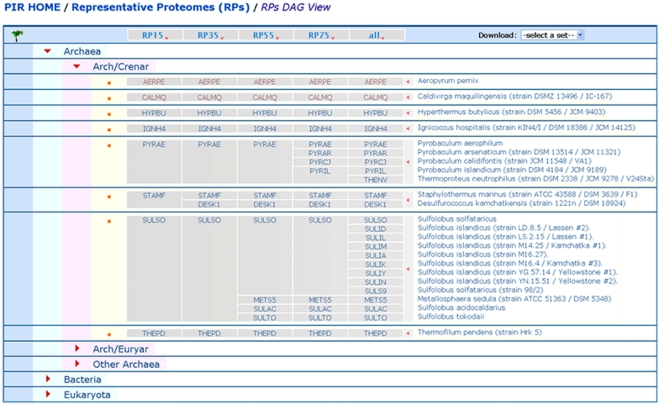
Browsing the Representative Proteome Groups (RPGs) and Representative Proteomes (RPs) at different thresholds.

The RPs at the four different thresholds can be viewed (Figure 4) at http://proteininformationresource.org/cgi-bin/rps_tree.pl. The top most nodes are Archaea, Bacteria and Eukaryota and the fully expanded view shows all the proteomes that have been analyzed to identify the RPs. Browsing the RPs at different threshold for different taxonomy nodes can provide clues as to which CMT is best for a particular branch and how the RPs are distributed in the taxonomy tree. Once a desired set of RPs is displayed on the screen, it can be printed for future reference.

### Data Update

The protein sets for the RPs and RPGs will be updated every four weeks. New proteomes will be added every six months. All releases will be archived.

## Discussion

In keeping the sequence analysis space both small and stable (that is, without exponential growth or major membership changes), the RPs offer several benefits. First, sequence searching and classification will be more computationally tractable. Second, manual curation can be more focused without having to deal with the moving target of rapidly accumulating protein sequences. Third, a standard set of Representative Proteomes (RPs) would enable the scientific community to make direct comparison between clusters and annotations produced by various methods. The ability to make direct comparisons, in turn, would provide a fourth benefit: to facilitate data and information integration.

The Representative Proteome datasets were created to provide the best possible coverage of protein information content while both reducing the number of sequences and providing a more even sampling of sequence space. Using the RP set of sequences increases the speed of similarity searches, and aids in the identification of homologs, protein family classification, and comparative genomic and proteomic analyses. The potential time savings of using a representative dataset such as RP are clear, but how does this translate to how one might use the database for similarity searches? We envisage that similarity searches would become a two stage process, whereby if one were to match a sequence in RP, as they are preferentially selected/ranked for biochemical characterization, then a hit against RP is likely to provide useful functional annotation and provide a good measure of the taxonomic distribution of similar sequences. However, if a hit against RP is not found, a full database search would be performed to ensure that potential hits are not missed. We have experimented with proteome groups at lower and higher identity threshold but our empirical tests show that a 55% CMT produce a relatively stable set of proteome groups that roughly follow standard taxonomic classifications (except for known taxonomic misclassifications) (Figure 2). We calculated how taking a purely taxonomic approach would affect the evenness of sequence space representation. The calculation is based on how often a given representative would represent a species/strain/isolate (hereafter “organism”) from a different genus, and also how often different organisms from an identical genus can be found in different representative groups. The first type of grouping occurs when two organisms from different genera are relatively close in sequence. Using a taxon-based approach, such closely-related organisms would both be representatives, and thus would in essence over-represent the encoded proteins. The converse is true when considering the split genera. Organisms from the same genus would collapse under one representative even though the sequence similarity warrants that they both be represented. We would effectively under-represent the proteins within. At a 55% CMT, we find that about 20% of the RPGs would differ from the current set if a purely genus-based approach were taken rather than the sequence-based approach used here (data not shown). This means that the resulting RPs would unevenly represent sequence space in 20% of the cases, with some related sequences being over-represented and others being under-represented. The opposing interpretation is that the sequence-based approach fails to accurately reflect taxonomy. Despite this, we favor the sequence-based approach because RPs are intended to both reduce and reflect sequence space.

Further justification for our approach can be obtained by examining the small number of groups that do not match taxonomic classification. Closer inspection of the groups where the same species are split into multiple RPGs reveals that nine out of these eleven species actually co-occur with related sub-species/strains. There is only one species that groups with another species instead of grouping with one of its own: (*Chlorobium phaeobacteroides (strain DSM 266)* is grouped with *Chlorobium limicola* (strain DSM 245/NBRC 103803) instead of with *Chlorobium phaeobacteroides* (strain BS1)). The classification of *Chlorobium* species is a contentious issue [13], with the issues being paralleled in our grouping, highlighting that *Chlorobium phaeobacteroides* (strain BS1) possibly requires re-classifying. Another mismatch situation occurs when a group has several genera. An example of such a RPG is the *Escherichia coli* group, which has proteomes from *Salmonella* and *Shigella*. Again this is not surprising as these classifications are based on differences other than at the molecular level, such as clinical reporting/manifestations [14], [15].

A comparison of our RPs with the proteome sets for the Quest For Orthologs (QFO) [5] shows that there are six proteomes that are not RPs but are on the QFO list. The QFO list is based on a taxonomy coverage consideration and did not include any empirical comparison of proteomes. Examination of these six proteomes to understand why they are not RPs shows that there were other proteomes (at the strain level) with higher proteome characterization scores. *Methanosarcina mazei* (NCBI taxonomy identifier: 2209), *Chlamydia trachomatis* (NCBI taxonomy identifier: 813), *Pseudomonas aeruginosa* (NCBI taxonomy identifier: 287), *Aspergillus fumigatus* (NCBI taxonomy identifier: 5085), *Halobacterium salinarium* (NCBI taxonomy identifier: 2242) and *Dictyoglomus thermophilum* (NCBI taxonomy identifier: 309799) have higher Proteome Priority Scores (score mostly based on the number of publications; see Materials and Methods) than *Methanosarcina acetivorans* (NCBI taxonomy identifier: 2214), *Chlamydia trachomatis* (NCBI taxonomy identifier: 315277), *Pseudomonas aeruginosa* (NCBI taxonomy identifier: 381754), *Aspergillus fumigatus* (NCBI taxonomy identifier: 451804), *Halobacterium salinarum* (NCBI taxonomy identifier: 478009) and *Dictyoglomus turgidum* (NCBI taxonomy identifier 515635).

Future improvements include the addition of viral proteomes to the RPs. However, the small size of many viral proteomes means that they are not amenable to grouping them in the same way as used in this study. Therefore, we plan to use the curated UniProtKB viral reference proteomes, a new and ongoing initiative to define viral reference strains for each virus genus (http://www.uniprot.org/news/2010/07/13/release).

In addition to being useful for curation purposes, RPs can also be used to speed up similarity searches, without significant loss of hit information. Furthermore, as the hits can be readily arranged in terms of taxonomy, RPs could be used in the analysis of large metagenomic datasets, such as those found in UniMES {Consortium, 2010 #5}. With such samples the protein sequences are usually fragments and the source organism is unknown. Consequently, there are two common issues that a search is trying to address: 1) Determine the full-length sequence that the fragment has come from, thereby allowing the identification of the full domain/functional repertoire likely to be present in the sample; and 2) Estimate the taxonomic composition of the sample. Taking the highest scoring match to RP will, on the whole, give an answer to both questions as the source organism in the target database is known and RP most of the time contains the best functionally characterized sequences. Using the RP will therefore provide rapid and reliable information about potential functions and likely taxonomic distributions found within the analyzed dataset.

## Supporting Information

Table S1 List of characterized proteins.
(TXT)

Table S2 Statistics on the cluster sizes for all Representative Proteome sets.
(XLS)
